# Moving Towards a Finer Way of Light-Cured Resin-Based Restorative Dental Materials: Recent Advances in Photoinitiating Systems Based on Iodonium Salts

**DOI:** 10.3390/ma13184093

**Published:** 2020-09-15

**Authors:** Monika Topa, Joanna Ortyl

**Affiliations:** 1 Faculty of Chemical Engineering and Technology, Cracow University of Technology, Warszawska 24, 31-155 Cracow, Poland; 2 Photo HiTech Ltd., Bobrzyńskiego 14, 30-348 Cracow, Poland

**Keywords:** photopolymerization, light-cured composites, cationic photoinitiator, free radical photoinitiator, iodonium salt, polymerization shrinkage

## Abstract

The photoinduced polymerization of monomers is currently an essential tool in various industries. The photopolymerization process plays an increasingly important role in biomedical applications. It is especially used in the production of dental composites. It also exhibits unique properties, such as a short time of polymerization of composites (up to a few seconds), low energy consumption, and spatial resolution (polymerization only in irradiated areas). This paper describes a short overview of the history and classification of different typical monomers and photoinitiating systems such as bimolecular photoinitiator system containing camphorquinone and aromatic amine, 1-phenyl-1,2-propanedione, phosphine derivatives, germanium derivatives, hexaarylbiimidazole derivatives, silane-based derivatives and thioxanthone derivatives used in the production of dental composites with their limitations and disadvantages. Moreover, this article represents the challenges faced when using the latest inventions in the field of dental materials, with a particular focus on photoinitiating systems based on iodonium salts. The beneficial properties of dental composites cured using initiation systems based on iodonium salts have been demonstrated.

## 1. Introduction

Nowadays, the most modern technologies for the production of polymeric materials are based on photochemically initiated processes. The synthesis of polymeric materials carried out by photopolymerization is one of the most efficient methods, thanks to which it is currently a very widespread and dynamically developing technique [1,2,3,4,5]. Compared to other methods, photopolymerization is considered environmentally friendly due to its low energy consumption, no use of solvents, and high speed at ambient temperature. In industrial practice, two types of photochemically initiated polymerization are most commonly used, namely radical and cationic polymerization [6,7,8,9]. Due to the presence of oxygen inhibition in the case of free radical photopolymerization, much attention is currently paid to the cationic, thiol-ene, and hybrid photopolymerization processes [10,11].

Polymerization using light, mainly ultraviolet (UV) light, was initially used in the coating industry, especially in varnishing for solvent-free paints and varnishes for the furniture and automotive industries [11]. Achieving high polymerization rates in fractions of seconds, resulting from the rapid formation of radicals or initiating ions, allows for high throughput of the production line [12]. Besides, the possibility of conducting photopolymerization processes at ambient temperature prepares polymeric materials carried out by photoinduced polymerization process one of the most efficient photochemical technologies. Currently, this type of polymerization is also used in many other industries, namely in photolithography for the production of printed circuits, in micro-replication for the production of spherical lenses, for photo curing polymeric adhesives, and in microelectronics for encapsulating integrated circuits [13]. The dynamically developing printing industry is a different direction of the application of photopolymerization, which enables printing on plastic or metal materials. Moreover, in recent years, a particular emphasis has been put on the use of photopolymerization processes for 3D-printing technology [14,15,16,17,18,19,20,21,22,23,24,25,26,27,28,29,30,31,32,33], including stereolithography in the design and formation of three-dimensional models [34].

All this means that not only has recently been an astonishingly rapid growth in the applications of technologies based on photopolymerization processes, but also the development of new materials determining the pace of this development [35]. Dynamic progress in the field of chemistry and technology of photoinitiated processes leads to the emergence of more and more sophisticated solutions in this field, an example of which may be successively developed new generation monomers [36,37], new, more effective photoinitiation systems [38,39,40] or new light sources [1,29] and methods of monitoring the online polymerization processes [41,42,43,44].

Photopolymerization processes play an increasingly important role in biomedical applications, for instance, in obtaining hydrogel polymer materials [45,46,47,48,49,50,51,52,53,54] or in vivo photocurable dental composites [55,56,57,58,59,60,61,62,63,64,65,66,67,68,69,70,71,72,73]. Applying photochemically initiated polymerization for obtaining dental polymer composites enables the use of unique and innovative features. The most important are:

- Short time of monomer/filler compositions curing (up to a few seconds);
- Conducting the reaction at room temperature;
- Low energy consumption;
- Spatial resolution (polymerization only in irradiated areas).

**Figure 1:**
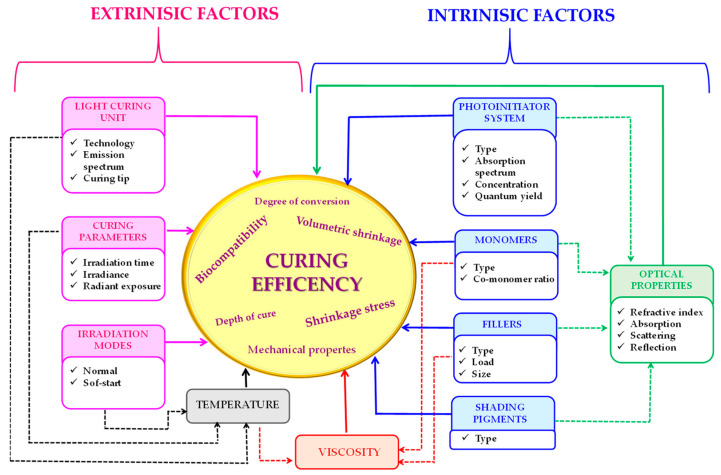
Illustrative diagram showing the influence of factors on the quality of obtained dental composites.

Nevertheless, obtaining polymer composites of the demanded properties, that is, above all, of favorable mechanical properties and reduction polymerization shrinkage, is still a significant challenge for the researchers. This is because many different factors, such as the selection of appropriate monomers, initiators, inorganic fillers, photopolymerization process time range, or the source and power of a light source, influence the quality of the composite obtained (Figure 1) [74].

Recently, iodonium salts have become of particular interest and are used as a component of initiating systems for the preparation of dental composites. This is directly due to the relatively good solubility of these salts in non-polar monomers. In addition, this group of compounds photodissociate with high initiation efficiency are thermally stable and show long-term stability under storage conditions [75,76].

In this paper, we aim to present commonly used monomers and photoinitiating systems for the photocurable dental composites and indicated their main disadvantages. Recent developments and progress in the future of photocurable resins have also been shown. A particular emphasis was placed on novel photoinitiating systems containing iodonium salts applied in dental adhesive resin.

## 2. Monomers Used for the Production of Dental Composites

**Figure 2:**
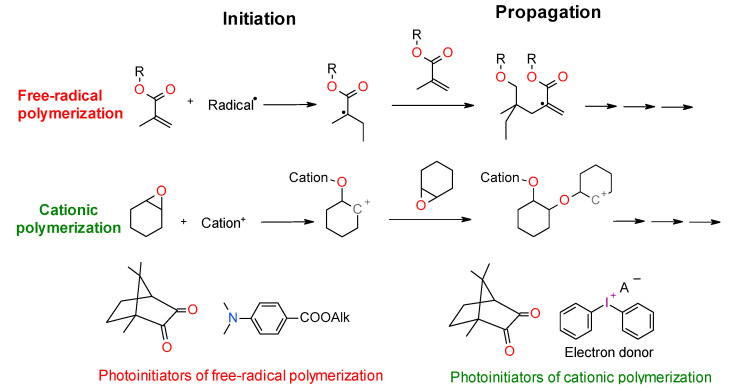
Mechanism of free radical polymerization and cationic ring-opening polymerization with their corresponding photoinitiation systems.

The mechanism of photopolymerization depends on using monomers. There are two main types of photopolymerization: radical polymerization and cationic polymerization (Figure 2). The type of organic matrix has a considerable impact on the properties of dental composites. It primarily affects mechanical strength, sorption, solubility, polymerization shrinkage, abrasion resistance, color stability, and biocompatibility [74,77]. Generally, the organic components of a typical photocuring composition constitute about 10–30% wt. [78]. The remainder is inorganic filling in the form of microparticles (≥0.4 μm) or a mixture of micro and nanoparticles (50 nm > 400 nm) [78]. In addition to photoinitiators, adhesion promoters and possibly antibacterial compounds are also added to dental composites [79,80,81].

### 2.1. Monomers for Free Radical Photopolymerization Processes in Dental Adhesive Resin Application

The most popular materials for obtaining dental composites through photopolymerization are (meth)acrylate monomers (RCB—resin-based composites) characterized by high reactivity, which form an organic matrix [82,83,84]. They guarantee obtaining networks with a high degree of crosslinking [84,85]. By free radical polymerization of the matrix monomers, a three-dimensional network is formed. Among the currently available dental composites, the most common are 2,2-bis[4-2-hydroxy-3-methacryloyloxypropyl)phenyl]propane (BisGMA) and triethylene glycol dimethacrylate (TEGDMA) [86].

**Figure 3:**
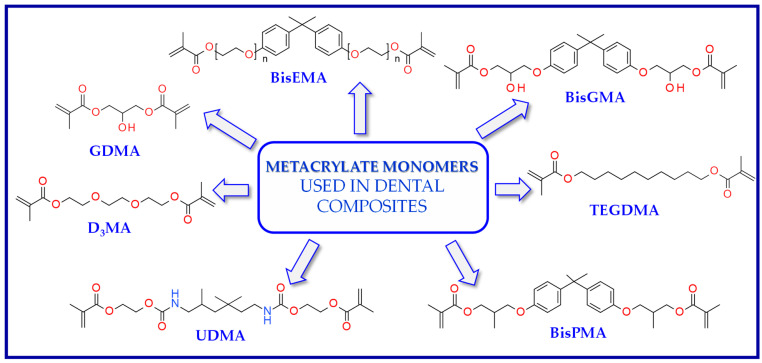
Examples of methacrylate monomers used in commercial and conventional dental composites based on free radical photopolymerization mechanism.

The use of BisGMA in dental materials, due to the presence of the aromatic structure of Bisphenol A in the core of the molecule, ensures low volatility of the composition and high modulus of the light-cured composite [87,88,89]. In turn, the use of the low-viscousity TEGDMA monomer, which is the so-called active diluent, allows the introduction of an appropriate amount of inorganic filler [90]. The weight proportions of both monomers are usually 7/3 or 8/2, where BisGMA is the main component. Another commonly used acrylate monomer is 1,6-bis-[2-methacryloyloxyethoxycarbonylamino]-2,4,4-trimethylhexane (UDMA) [91]. The content of rigid urethane groups guarantees dental composites with favorable strength properties [92,93,94]. In addition to the aforementioned BisGMA, TEGDMA, and UDMA, other common dental monomers polymerized via the free radical process are also ethoxylated BisGMA (BisEMA). This monomer is used for reducing water absorption by the organic matrix. In addition, the lack of -OH also causes this monomer to be less viscous than BisGMA. An array of monomer structures for the base dimethacrylate materials, as well as new monomers, are given in Figure 3 [95,96,97,98,99,100,101].

All dental composites based on crosslinking dimethacrylates exhibit an inherent problem of 2–14% volumetric shrinkage during the photopolymerization process [102]. These stresses may produce defects in the composite–tooth bond, leading to bond failure, microleakage, postoperative sensitivity, and recurrent caries. Such shrinkage stresses could also cause deformation of the surrounding tooth structure when the composite–tooth bond is strong, predisposing the tooth to fracture [103]. The polymerization shrinkage of low molecular monomers is more pronounced when compared to that of high molecular monomers; however, high molecular monomers are very viscous (Table 1). For these reasons, polymerization shrinkage is dictated by a complex interplay among resin viscosity, polymerization rate, degree of conversion, and network structural evolution, where each of these properties cannot be individually manipulated and studied without having a significant impact on other properties.

Moreover, due to the inhomogeneous network architecture, which is obtained during a free radical photopolymerization process, the final materials tend to show a somewhat brittle behavior, and the occurring shrinkage stress could lead to delamination, deformation or mechanical failure of the final composites materials. The observed shrinkage stress evolves during polymerization reaction upon transitioning of the applied formulation from the liquid to solid-state (i.e., gel point) and is built up upon vitrification until the final conversion is reached. Before free radical photopolymerization, the monomers are situated at van der Waal’s distance towards each other (approximately 3.4 Å) [104]. The occurring shrinkage stress upon gelation is partially due to the formation of covalent bonds between the respective monomers, where the revealing distance is only 1.5 Å [105]. Incomplete free radical photopolymerization, volumetric shrinkage, and stress are some of the primary disadvantages of current methacrylates resin-based dental composites. Generally, attempts to increase the double-bond conversion and reduce polymerization shrinkage and stress have been conducted [105].

### 2.2. Monomers for Cationic Photopolymerization.

In recent years, the application of ring-opening cationic photopolymerizable epoxy–monomer-based compositions for dental fillings have found increasing attention in different articles and patent applications [84,106,107].

**Figure 4:**
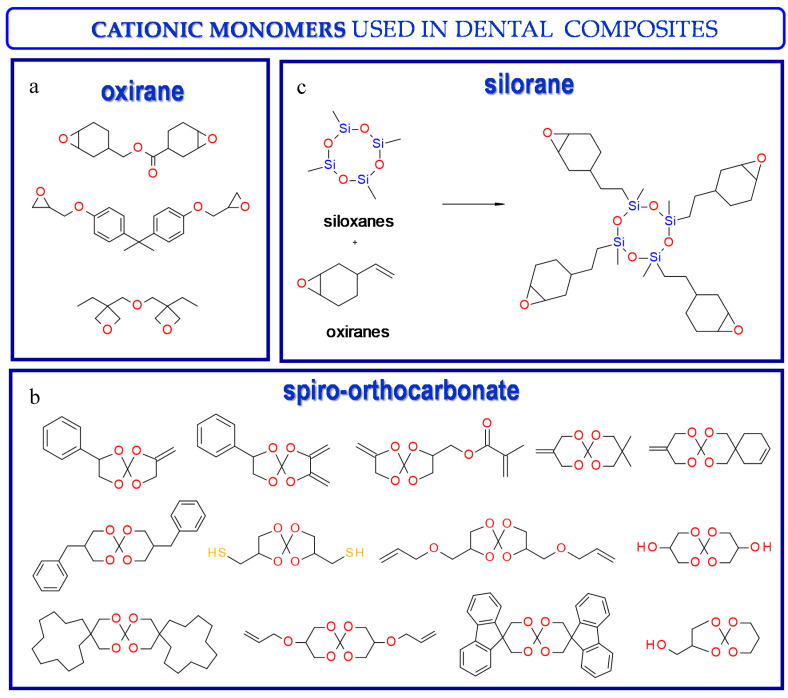
Examples of monomers used in cationic photopolymerization (**a**) oxirane, (**b**) spiro-orthocarbonate, (**c**) silorane: a merger of siloxanes and oxiranes.

Thus, based on the cationic photopolymerization process, new-generation photocuring dental materials, including oxiranes [84], siloranes [108], oxetanes, and spiro-orthocarbonate [109], were developed (Figure 4). Dental materials based on these monomers have achieved clinical success because they have significantly reduced polymerization shrinkage to below 1% and minimized polymerization stress compared to traditional methacrylate materials [106]. The mechanism of compensation for systolic stress in this system was achieved by the phenomenon of opening the oxirane rings during the cationic photopolymerization process, which proceeds with a small change in the volume of the system [110].

Crosslinking cycloaliphatic epoxy compounds were particularly of interest because they demonstrate significantly lower shrinkage than dental methacrylate resins (e.g., cycloaliphatic epoxide 3,4-epoxycyclohexyl-methyl-3,4-epoxycyclohexane carboxylate and the diglycidyl ether of bisphenol A, which improved the mechanical properties of the cured composite). Moreover, these epoxy resins were reactive enough to be cured by cationic photopolymerization in an acceptable time frame and to an adequate depth using a dental Vis-LED light source. In addition to epoxy resins, oxetanes were evaluated for dental applications [111]. The reactivity of oxetanes is mainly controlled by the ring stress and the basicity of the ring oxygen. However, oxetanes demonstrate higher basicity. The ring-opening cationic photopolymerization of oxetanes was also characterized by a significantly lower shrinkage in comparison to methacrylates. From the investigated oxetanes, the hydroxy group containing monomer possessed the highest polymerization rate [112].

In turn, spiroorthoesters (SOEs) and spiroorthocarbonates (SOCs) (Figure 4) are other monomers that are polymerized via to the cationic mechanism and are increasingly used in dental applications. Spiroorthoesters (SOEs) and spiroorthocarbonates (SOCs) are the most widely studied expanding monomers. SOCs are double-cyclic acetals that polymerize under acidic catalysis but are stable under basic conditions. When these compounds polymerize by double ring-opening photopolymerization (ROP), poly(ether-carbonates) are produced. In general, bi-cyclic compounds cured by ROP shrink less as they harden because of an increase in the excluded free volume associated with the ring-opening process. Bailey [113] investigated bi-cyclic compounds, such as spiro-orthocarbonates (SOCs), that can be used as an expanding co-monomer in RBC formulations. Ring-opening reactions with SOCs produce expansion (3.5%), which could counteract normal shrinkage [114]. However, SOCs exhibit incomplete ring-opening, as well as limited solubility and minimal copolymerization in dimethacrylate resins, resulting in minimal shrinkage reduction.

Nevertheless, compared to traditional composite materials, spiroorthocarbonate-based composites show less polymerization shrinkage and twice as much adhesion to enamel [109].

However, the most recent modification on the polymer matrix is based on using ring-opening polymerization of the silorane molecules, instead of free radical polymerization of dimethacrylate monomers [115]. They are built of a siloxane backbone, which gives them hydrophobic and cycloaliphatic oxirane molecules responsible for low polymerization shrinkage. These monomers have provided particularly interesting and commercially viable results. Such monomers “open” their molecular structures with local volumetric expansion, and this may partly or totally compensate for volumetric shrinkage from C=C or similar polymerization [116,117]. Based on the literature reports, the use of siloranes has been shown to guarantee a reduction in the polymerization shrinkage to 0.94% [108].

Examples of monomers that polymerize thorough to the cationic mechanism and have reduced polymerization shrinkage are shown in Figure 4.

**Figure 5:**
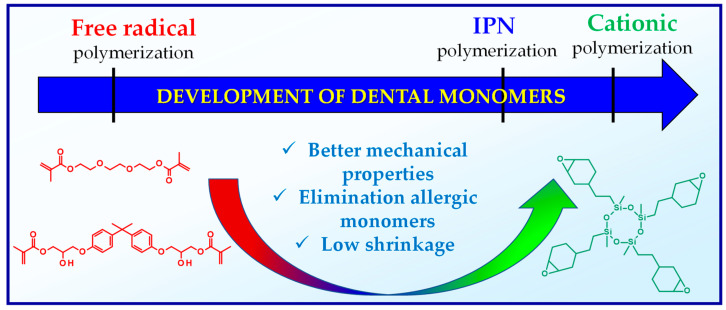
Comparison of the properties of monomers polymerizable via free radical mechanism with monomers polymerizable via cationic mechanism.

The development of new monomers polymerizing via the cationic mechanism contributed to a significant reduction in the polymerization shrinkage of dental composites and obtaining dental composites with better mechanical properties. Moreover, acrylate monomers, which often cause severe allergies, have been eliminated (Figure 5).

## 3. Commonly Used Photoinitiating Systems for Dental Application

**Figure 6:**
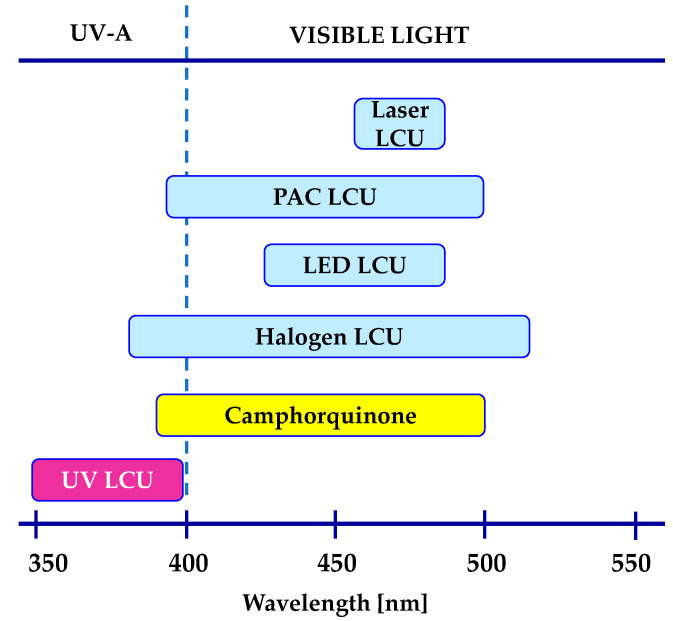
The range of emission spectra of UV and visible light-curing units and the range of the absorption of standard co-initiators camphorquinone used in the dental application (UV—ultraviolet, LCU—light-curing units, LED—light-emitting diode, PAC—plasma arc).

Photoinitiating systems for obtaining dental composites are particularly important. They affect such parameters as the efficiency of the photopolymerization process and the choice of a light source (Figure 6).

**Figure 7:**
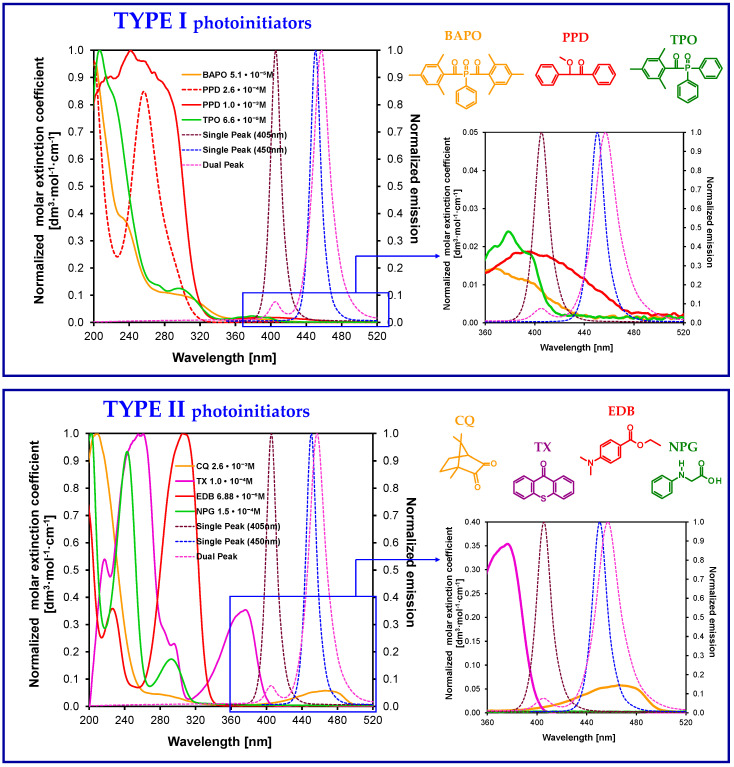
Comparison of the normalized molar extinction coefficient of standard type I initiators (top) and type II initiators together with amines (bottom) used in dental applications with the emission characteristics of standard light-curing units.

To date, several initiation systems for radical photopolymerization processes have been developed. In Figure 7, the absorption spectra of standard initiators in comparison with the emission characteristics of the commonly used light-curing units are presented. 

### 3.1. Bimolecular Photoinitiator System Containing Camphorquinone and Aromatic Amine

The commonly used photoinitiating system for the radical photopolymerization process for dental composites is the system based on camphorquinone/amine. Widely used camphorquinone (CQ) is a diketone that absorbs radiation in the range from 200 to 300 nm, which corresponds to the π-π^*^ transition; however, this band is not useful for applications in vivo photocuring of dental materials. The second absorption range of camphorquinone is located in the visible light range from 400 to 500 nm (Figure 7), where the band is responsible for the transition of the n-π^*^ carbonyl group. The presence of a long-term absorption band means that this compound has been used as a component of initiating systems, mainly free radical photopolymerization. Nevertheless, for camphorquinone, the value of the molar extinction coefficient in the range of 400–500 nm is only 40 [dm^3^·mol^−1^·cm^−1^] [118,119]. Therefore, in the case of initiating systems based on CQ, a significant part of the energy emitted by Vis-LED light sources (emission range 420–515 nm) is lost. Therefore, the efficiency of polymerization of standard methacrylate dental materials in the presence of the only camphorquinone is insufficient.

Moreover, the addition of primary amines to the polymerization system does not significantly accelerate the radical photopolymerization process. However, the polymerization rate significantly increases when tertiary amines as co-initiators of radical photopolymerization are used. Amines such as ethyl-4-dimethylaminobenzoate (EDAB/EDMAB), 2-(dimethylamino)ethyl methacrylate (DMAEMA), N,N-dimethylptoluidine, N-phenylglycine (NPG), dimethylbenzoate are used as photosensitizers for CQ [120,121,122,123,124,125,126,127]. However, among these compounds, ethyl-4-dimethylaminobenzoate (EDB) is the most popular co-initiator in dental materials due to its high efficiency and low basicity. 

**Figure 8:**
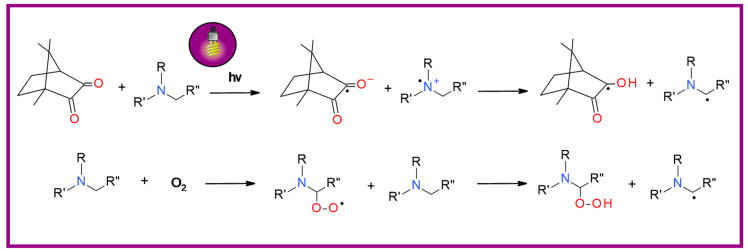
The initiation mechanism using bimolecular photoinitiator system containing camphorquinone and aromatic amine; mechanism of reaction amine with oxygen.

In the step of generating radicals in the photolysis process, amine interacted with the excited camphorquinone molecule. This process involves the transfer of the electron from the amine to the ketone, followed by the proton’s abstraction [128]. The radicals initiating the polymerization process are mainly radicals formed from amines. Another mechanism that affects the amine’s efficiency as a co-initiator is the formation of free radicals during the oxygen scavenging reaction. Oxygen present in the monomer can react with amines to form a peroxide radical. This, in turn, can react with another amine to release a new free radical. In this way, the inhibitory effect of oxygen is weakened. The mechanism amine, with CQ and oxygen, is presented in Figure 8 [129,130].

**Figure 9:**
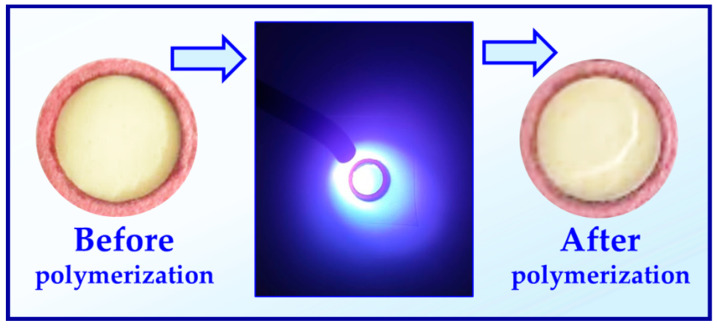
Color of a polymer composition composed of CQ (0.3 (wt.%)/EDB (0.5 wt.%) and BisGMA/TEGDMA (7:3) before and after photopolymerization.

**Figure 10:**
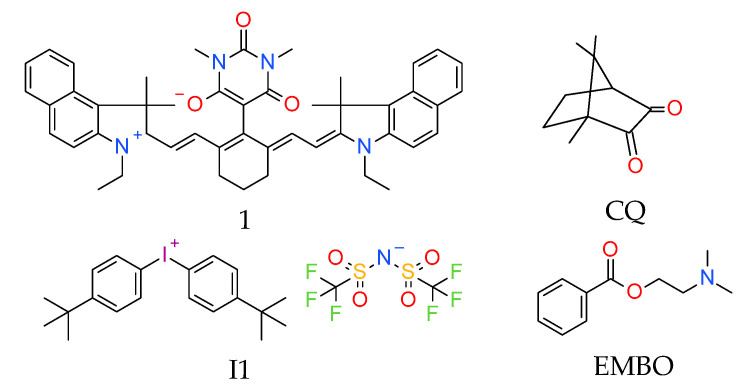
Structure of the photoinitiating system, based on CQ (0.3 (wt.%)/EMBO (0.5 wt.%) and (1) = 0.01 wt.%, (I1) = 4 wt.% or (1) = 0.02 wt.%, (I1) = 4 wt.%, which generates the brown color after photopolymerization.

Nerveless, the underlying problem of this system is the fact that a too high concentration of camphorquinone in dental composites may generate a yellow (Figure 9) or even brown color (Figure 10). Thomas Brömme et al. presented the initiating system in the form of iodonium salt bis(4-t-butylphenyl)iodonium bis(trifluoromethylsulfonyl)imide (I1), cyanines derivatives (1), camphorquinone (CQ) and dimethylamino ethylbenzoate (EMBO), which showed a brown color after light curing [131].

Such discoloration can influence the aesthetics and quality of the final product. A completely different problem of initiating systems based on camphorquinone and amine in composites (e.g., enamel–dentin adhesives or self-adhesive cement) containing monomers with carboxylic groups having acidic properties is because of the reaction of amines with these monomers. That, in turn, can contribute to amine consumption and decrease the efficiency of the initiating system. The limitations of amines, especially EDAB, include not only unstable in acidic conditions [132], unstable in acidic dental resin formulations [133,134], but also sensitivity to oxygen inhibition [135]. In addition, amines are a cytotoxic and genotoxic factor [101].

### 3.2. 1-Phenyl-1,2-propanedione as an Effective Alternative Photoinitiator

1-phenyl-1,2-propanedione (PPD) [136,137] as a Norrish type I photoinitiator, reacts by photolysis, where the cleavage of the C–C bond between the carbonyls functional groups of its molecule leads to the formation of free radicals starting the polymerization. However, PPD can also react via a co-initiator, since it bears the same diketone group as camphorquinone. Then, radicals derived from the amine-based co-initiator H-transfer are responsible for starting the polymerization. Therefore, PPD is an alternative to camphorquinone/amine systems initiating radical photopolymerization processes. The research proved that the higher mechanical properties of the model resin composite containing PPD compared with that containing CQ are obtained [138,139]. Moreover, the study shows that PPD is useful not only for photosensitizers but also for photocrosslinking agents for dental composite resins with similar efficiency to CQ [140]. 

Besides, PPD has reduced properties associated with the yellowing effect, which results directly from its absorption characteristics, which is mainly in the UV-A range and goes to the visible range with the slope of the absorption band. For dental composites containing a PPD initiator, in order to achieve conversion rates compared to those of the camphorquinone/amine system, it is necessary to use LED light sources with two violet emission bands (380–420 nm and blue 420–520 nm) (Figure 7, Table 2). However, not all dental photocuring lamps guarantee such emission characteristics; therefore, the use of PPD initiator composites requires the use of dual-peak LEDs.

### 3.3. Phosphine Derivatives as Free radical Photoinitiators for in Visible Light Cure Polymerization

Mono-acylphosphine oxides (MAPO) and bis-acylphosphine oxide oxides (BAPO) are mainly photoinitiators used in a dental application that absorb in the 380–450 nm range. One of the first commercially available mono-acylphosphine initiators is diphenyl (2,4,6-trimethylbenzoyl)phosphine oxide (TPO). This initiator is known on the market as Lucirin^®^ TPO. The conduct of its photopolymerization follows an α-cleavage mechanism, in which TPO undergoes hemolytic α-cleavage of the carbon–phosphorus bond and generates two free radicals, (Table 2) both capable of initiating polymerization [141,142,143,144].

These initiators show the high efficiency of generating radicals; however, their disadvantage is the absorption characteristics, which is mainly located in the UV-A range, and the effective use of their absorption characteristics occurs when dual peaks LED lamps are used. This initiator absorbs in the range of only 350–380 nm. Nevertheless, Pedro Paulo A.C. Albuquerque et al. [145] showed that using a photoinitiator system containing TPO might improve the color stability of resin composites compared with the traditional CQ/amine system while attaining similar physicochemical properties for the composite. In particular, unlike the Q-based systems, TPO does not require the use of an amine co-initiator [141,142,143,146] so that the polymerization is not negatively affected by the acidic environment like itself-etch adhesives.

The other phosphine derivative is bis(2,4,6-trimethylbenzoyl)phosphine oxide (BAPO). This photoinitiator is a promising alternative to initiating free radical photopolymerization upon halogen light to obtain dental resin. Remarkably, the efficiency of photoinitiating is similar to the conventionally used initiating system (CQ + EDAB). Additionally, the supplement of iodonium salt or amine can improve the effectiveness of these systems [147].

Moreover, the BAPO and TPO revealed concentration-dependent cytotoxic effects in human oral keratinocytes and V79 cells. However, in contrast to CQ, no generation of intracellular reactive oxygen/nitrogen species (ROS/RNS) was found. Only BAPO induced genotoxicity in V79 cells [148].

### 3.4. Germanium Derivatives—Extending the Scope of Visible Light Photoinitiators

**Figure 11:**
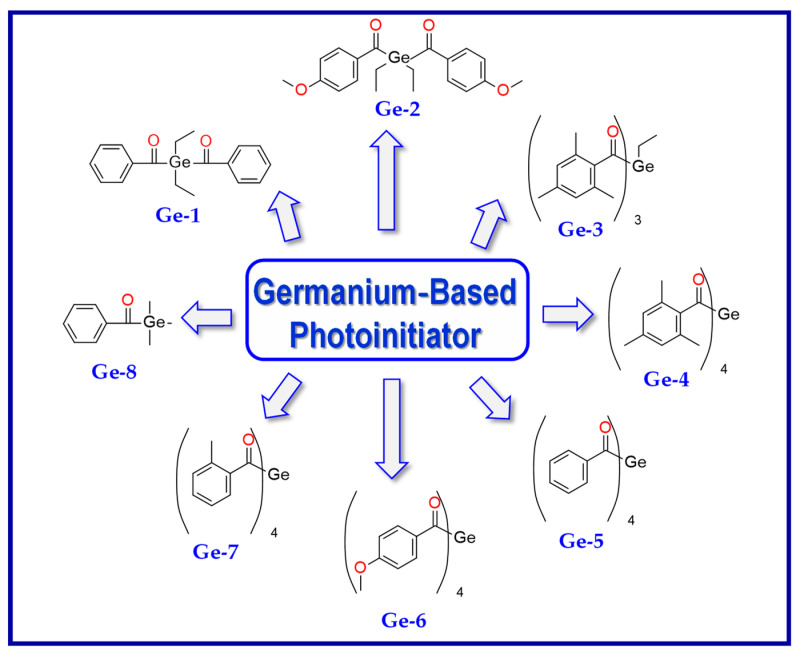
Structure of novel germanium derivatives.

Much progress was noted when Liska et al. developed visible light photoinitiators based on germanium compounds. For the first time, they showed that germanium compounds such as benzoyltrimethylgermane (Ge-1) or dibenzoyldiethylgermane (Ge-2) (Figure 11) represent efficient visible light photoinitiators for methacrylate monomers [149,150,151]. In contrast to Lucirin^®^ TPO (λ_max_ = 385 nm), Ge-1 (λ_max_ = 411 nm) and Ge-2 (λ_max_ = 418 nm) show a pronounced redshift in their absorption. It means that they absorb light more strongly within the visible region.

**Figure 12:**
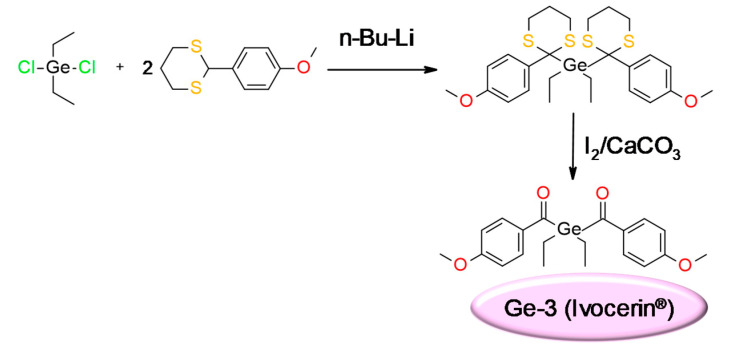
Synthetic pathway to obtain Ivocerin^®^ product.

Based on the results of these mechanistic investigations and the evaluation of different synthesis methods and structural variations of germanium compounds, bis-(4-methoxybenzoyl) diethyl-germane (Ge-3) was selected as the appropriate photoinitiator and protected by a patent under the name of Ivocerin^®^. Furthermore, this initiator showed no cytotoxicity. The synthesis of this compound is shown in Figure 12.

**Figure 13:**
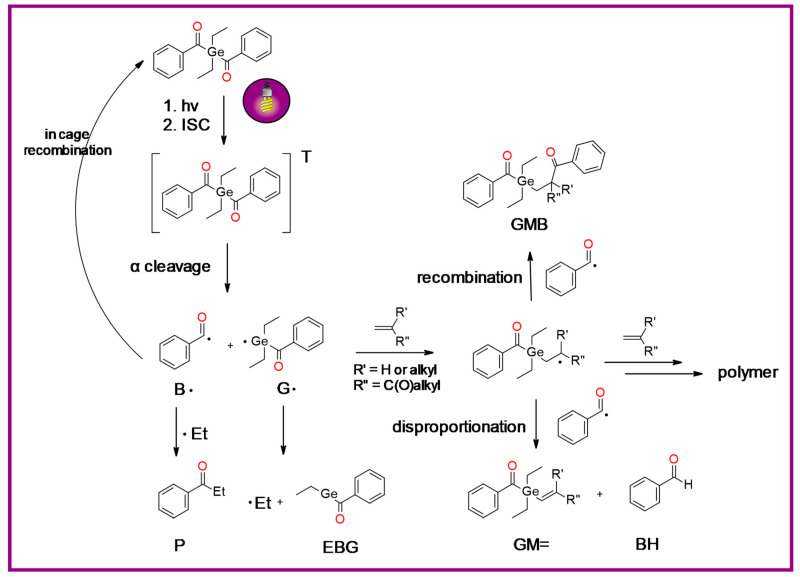
Proposed reaction mechanism of germanium derivatives as a photoinitiator in the presence of monomers.

In addition, germanium derivatives exhibit quick curing and excellent bleaching behavior. The proposed reaction scheme of germanium derivatives as a photoinitiator in the presence of monomers is presented in Figure 13 [151]. They require a much lower concentration of photoinitiator to achieve comparable mechanical properties than commonly used photoinitiators. However, the main limitation of these systems is that they are active initiators for free radical polymerization [152], but they do not guarantee cationic polymerization initiation.

### 3.5. Hexaarylbiimidazole Derivatives

**Figure 14:**
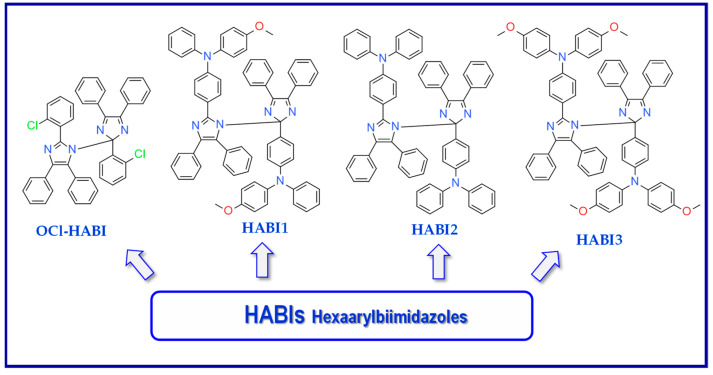
Structures of different hexaarylbiimidazole (HABI) derivatives.

Hexaarylbiimidazoles (HABIs) were synthesized for the first time in 1960 by Hayashi and Maeda [153]. Figure 14 shows the structures of exemplary compounds, HABI derivatives [154].

**Figure 15:**
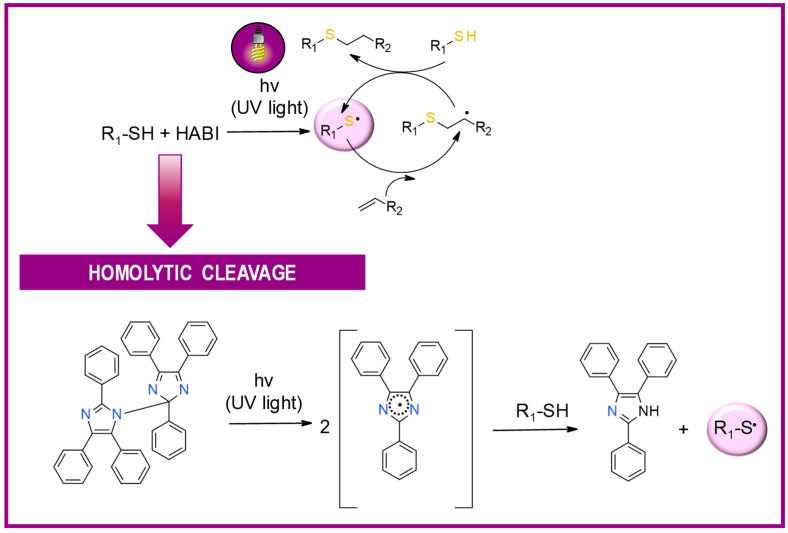
The radical-mediated thiol–ene polymerization mechanism.

HABI derivatives are usually used for thiol-en systems. In turn, thiols are commonly used as co-initiators in combination with hexaarylbiimidazoles [155]. After irradiation, the binding between imidazole HABI undergoes homolytic cleavage, generating two relatively stable, long-lived lophyl radicals that are unreactive with oxygen and show slow recombination rates [156], attributable to steric hindrance as well as electron delocalization [157,158]. Then, HABI-derived lophyl radicals abstract hydrogen from the thiol to generate initiating thiyl radicals (Figure 15).

**Figure 16:**
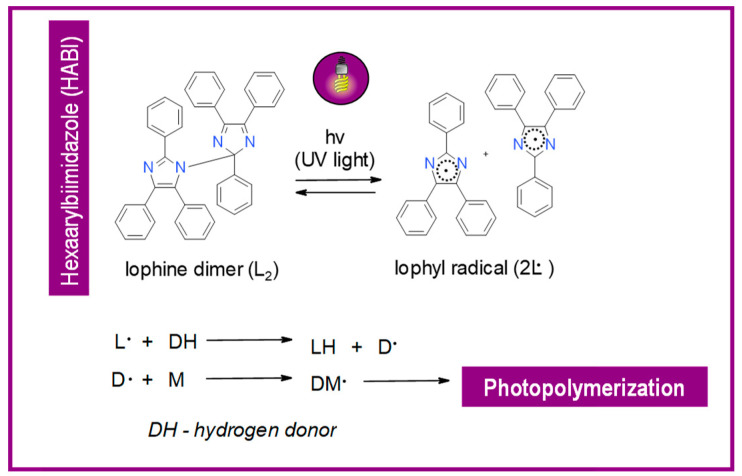
Proposed mechanism of the photolysis of the carbon-nitrogen (C–N) bond between the imidazole rings of HABI.

**Figure 17:**
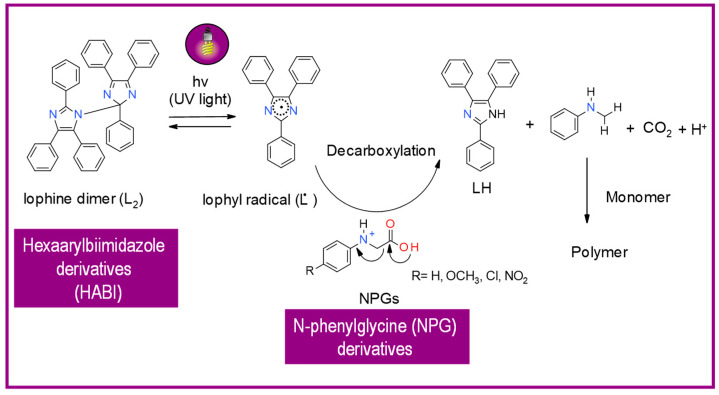
Photoinitiation process of type-II photoinitiator systems based on 2-chlorohexaarylbiimidazole (o-Cl-HABI) and various N-phenylglycine (NPG) derivatives.

Other mechanisms of photoinduced cleavage of photoinitiator, derivatives HABI have also been proposed. Their schemes with an explanation are presented in Figure 16 and Figure 17 [154,159].

Unfortunately, commercially available HABI derivatives have several significant disadvantages: poor absorption in the visible spectrum, sometimes requiring a photosensitizer; low solubility in standard resins used in a dental application; and low solubility in organic solvents [160,161]. Nevertheless, despite the relatively low absorption of visible light, HABI photoinitiators are useful in initiating thiol-ene photopolymerization processes.

### 3.6. Silane-Based Derivatives

Formable, soluble, and high molecular weight polysilanes are widely used as photoconductors, photoresist materials, and photoinitiators for free radical polymerization. These compounds have strong absorption in the 300–350 nm range [162]. Upon irradiation at this band, polysilane undergoes fast photodegradation yielding silylenes and silyl radicals. Research on polysilanes has been carried out in the last century. For example, West et al. [163] proved that these compounds are highly effective in free radical photopolymerization. West et al. also assumed that the phoinitiating process consists of a reaction of silyl radicals with vinyl monomers. It has also been proven that polysilanes of which iodonium salts [164] or pyridinium can be used for cationic photopolymerization of cyclic ethers (e.g., cyclohexene oxide) and vinyl ethers, (e.g., n-butyl vinyl ether).

Currently, a particular studied compound based on a silane derivative is tris (trimethylsilyl) silane (TTMSS). TTMMS was synthesized by Gilmanand et al. in 1965 [165]. Nearly 20 years later, Chatgilialoglu et al. proved that TMMS could be used as a radical reducing agent [166,167]. The TTMS radical is commonly used as a component in photoinitiating system [168,169,170,171,172,173,174] by Lalavée et al. They also reported that tris (trimethylsilyl) silane had the following attributes: a high inherent reactivity for the addition to double bonds, and also a low ionization potential (which is associated with an oxidation process and/or the formation of silylium cations). Currently, the photoinitiating system consisting of TTMS is reactive in free radical polymerization (FRP) [175] as well as in free radical-promoted cationic polymerization (FRPCP) [176]. Systems based on TTMSS have the ability for effective oxygen consumption. Therefore, it can overcome the classic and well-known inhibition of FRP or FRPCR by oxygen [169]. Moreover, TTMSS indicated no toxic reaction when tested in biological research [177]. Photoinitiating system based on TTMSS and other co-initiator such as benzophenone (BP), isopropylthioxanthone (ITX), camphorquinone (CQ) is highly reactive and even better than EDB using in dental composities [169]. Moreover, Song et al. in 2016 proved that TTMSS could be used as a substitute for amine-type co-initiator for free radical photopolymerization of methacrylate monomers used to obtain a dental composite.

In 2016, Mariem Bouzrati-Zerelli et al. developed an entirely new class of initiators, silyl glyoxylates (DKSi, Et-DKSi, Bn-DKSi), to free radical photopolymerization for obtained dental composites. Silyl glyoxylates are high-performance type I photoinitiators in the visible range. In combination with an appropriate amine, iodonium salt, or a phosphine, photopolymerization efficiency is improved. DKSi-based PIs outperformed the performance of the CQ to induce FRP under blue LED at 477 nm for thin (20 μm) and thick (1.4 and 6 mm) films. Excellent bleaching properties for this initiator were also observed [178].

**Figure 18:**
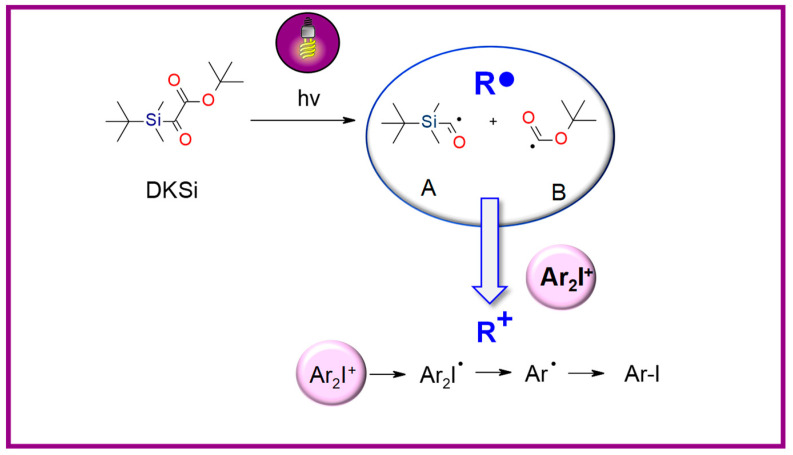
Proposed mechanism of formation cations from the photoinitiating system DKSi/iodonium salt.

Other articles have proven that the same silyl glyoxylate, combined with an iodonium salt, can be useful for initiating cationic photopolymerization and, thus, hybrid polymerization [179]. The proposed mechanism of formation cations from a two-component photoinitiation system based on DKSi and iodonium salt (Ar_2_I^+^) under irradiation is presented in Figure 18.

**Figure 19:**
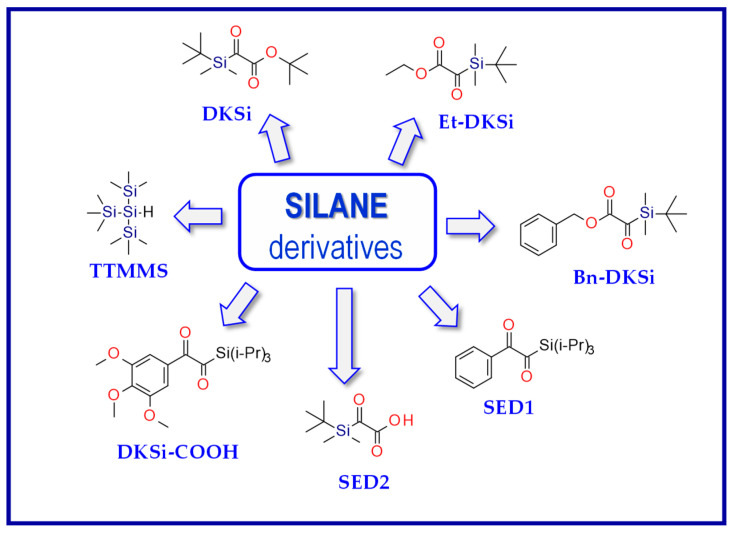
Chemical structures of the silane derivatives: TTMMS—tris (trimethylsilyl) silane; DKSi—tert-butyl (tert-butyldimethylsilyl) glyoxylate; Et-DKSi—ethyl (tert-butyldimethyl)silyl glyoxylate; Bn-DKSi—benzyl (tert-butyldimethyl)silyl glyoxylate; SED1—1-phenyl-2-(triisopropylsilyl)ethane-1,2-dione;SED2—1-(3,4,5-trimethoxyphenyl)-2-(triisopropylsilyl)ethane-1,2-dione; DKSi-COOH—2-oxo-2(tert-butyldimethylsilyl) acetic acid.

Kirscher et al. continued research on new silane derivatives, where the alkoxy group of the ester function of the previously developed silane derivatives was replaced, alkyl(trialkylsilyl)glyoxylate, by an aryl group to form 1-aryl-2-(triisopropylsilyl) ethane-1,2-diones (SEDs). Compared to CQ and DKSi, these compounds present shifted absorption spectra to longer wavelengths (λ_max_ = 486 nm for SED1 and 468 nm for SED2 in toluene). Therefore, they are suitable for free radical photopolymerization under air upon exposure to blue (@ 455 nm) and even green (@ 520 nm) LEDs. Additionally, just like DKSi, show photobleaching properties [180]. The same team modified the structure of DKSi, replacing the ester function of the DKSi with a carboxylic acid function to form 2-oxo-2 (tert-butyldimethylsilyl) acetic acid (DKSi-COOH). This way, DKSi-COOH, with its excellent bleaching properties, high water solubility, and excellent stability in acidic conditions, were obtained. Moreover, it is a photoinitiator that is useful for the free radical photopolymerization processes of BisGMA/TEGDMA monomers composition and leads to remarkably high polymerization performances in monomer under exposure to the LED at 477 nm [181]. The structures of the silane derivatives are presented in Figure 19.

### 3.7. Thioxanthone Derivatives (TX)

Some ionic derivatives of thioxanthone dyes are miscible with water and may constitute an attractive alternative to the photopolymerization of dental adhesive [182]. Derivatives of thioxanthone are type II bimolecular photoinitiators used for free radical and cationic photopolymerization [183]. Photoinitiation by thioxanthone derivatives is based on the reaction of their triplet excited states with the hydrogen donor, resulting in the formation of the initiating radical (Table 2). In turn, they suffer from a diffusion-controlled reduction of reactivity and deactivation by back electron transfer.

**Figure 20:**
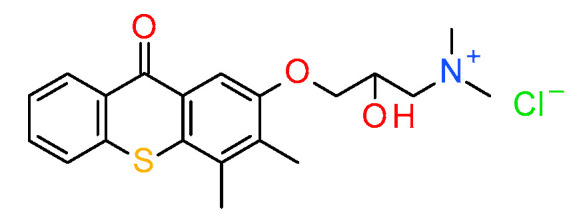
Structure of QTX.

Several articles on the use of thioxanthone as components of initiation systems for a dental application have been reported [184]. They are usually in two or three-component systems with co-initiators, e.g., an amine or an iodonium salt. The use of such a system leads to comparable conversion rates to the use of the CQ/aromatic amine system; however, the thioxanthone system is generally less reactive [184]. Ely et al. proved that the combination of an elastomeric methacrylic monomer used in a dental application and a water-soluble photoinitiator (2-hydroxy-3-(3,4-dimethyl-9-oxo-9H-thioxanthen-2-yloxy)-*N,N,N*-trimethyl-1-propanium chloride (QXT) (Figure 20) in a self-etching adhesive showed promising instant bond strength to dentin. Moreover, this composition can minimize the effects of concentration stress and phase separation in aquatic environments [151].

## 4. Onium Salts as an Innovative Component of Photoinitiating Systems for Photopolymerization Processes in Dental Applications

In recent times, onium salts, i.e., sulfonium and iodonium salts, particularly in the form of diaryliodonium salts, have been playing an increasingly important role in initiating photopolymerization processes [185,186,187,188,189,190,191,192]. 

All the properties of ionic compounds supporting their commercial use as photoinitiators depend only on their structure. It has been shown that the cation of iodonium salt, absorbing electromagnetic radiation, is responsible for the photochemical properties of these compounds as photoinitiators. Thus, the structure of the cation determines the initiator’s properties, such as the location of the maximum absorption (λ_max_), molar absorption coefficient (ε), the quantum efficiency of the initiator, and even thermal stability. On the other hand, the nature of anion has a decisive influence on the suitability of the initiating system as a photoinitiator. The type of anion determines the power of protic acid generated during photolysis, directly affecting the efficiency of initiation and the kinetics of the polymerization process. However, the essential properties of iodonium salts, from their applications in cationic polymerization processes (in addition to solubility in monomers) are their optical properties, i.e., the location of the maximum absorption (λ_max_) and molar extinction coefficient (ε) [75,76].

**Figure 21:**
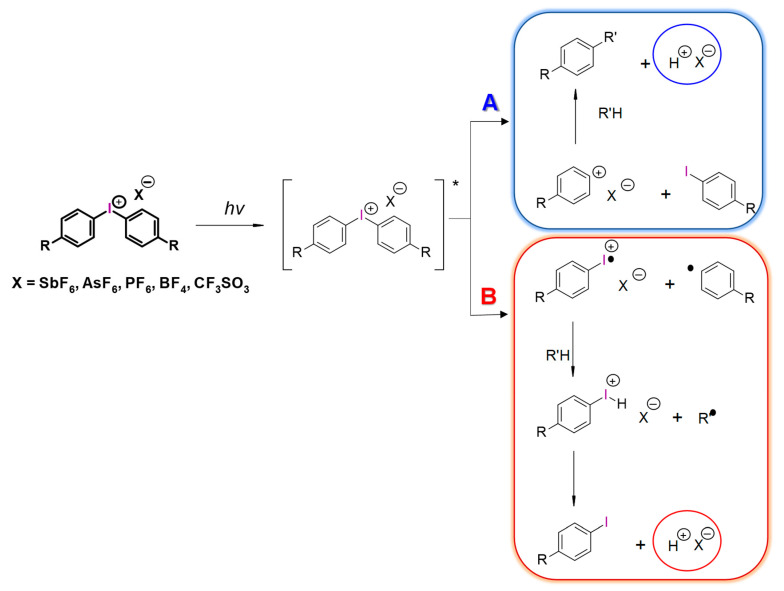
Photodissociation of iodonium salts by the mechanism: A—heterolytic and B—homolytic.

Diaryliodonium salts, with a weakly nucleophilic counter ion, are efficient photoinitiators for cationic photopolymerization. Due to the low C-I binding energy, which is 26–27 kcal/mol, after irradiation, diaryliodonium salts are broken down to a radical-cation, and reactive aryl radical and an anion [193,194,195] (Figure 21).

However, commercial iodonium salts currently used in the industry have light absorption characteristics in the UV-C range, i.e., λ_max_ = 220–280 nm, and that have very low or zero light absorption in the long-term UV-A range (λ > 300 nm). Table 3 shows the names and formulas of the commercially available iodonium salts along with their positions of the maximum absorbance.

Therefore, the light sensitivity of commercial cationic photoinitiators is in the short wavelength range of UV light, which is a significant technological problem in their different applications, as well as dental materials applications. 

**Figure 22:**
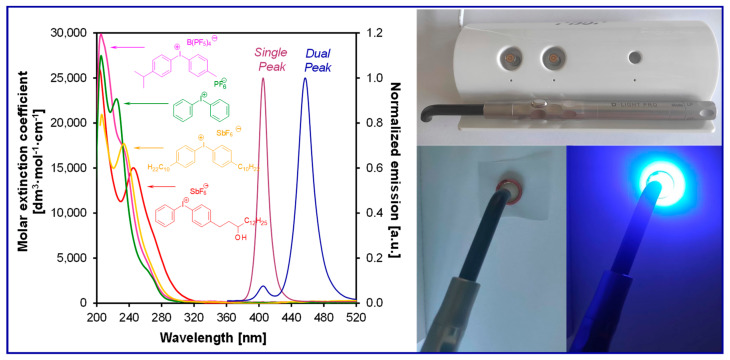
Comparison of the absorption characteristics of commercial iodonium photoinitiators with the emission characteristics of a commercially available dental lamp D -LIGHT^®^ PR and photograph of the lamp.

This is because the light sources used in dentistry usually have emission in the range from 420 to 515 nm (Single peak LCUs) (in modern lamps 380–440 nm) or possibly from 380 to 520 nm (Dual peak LCUs). The result is that commercially available cationic photoinitiators have mismatching of the absorption characteristics with the emission characteristics of these light sources (Figure 22). This makes them unsuitable for initiating photopolymerization processes in the UV-A range and in the visible area, which is a significant technological problem due to the low efficiency of the obtained polymer materials. Although this activation strategy is satisfactory in some applications, such as coating materials, the use of UV light is not recommended in the biological field. However, the use of absorbing dyes in the visible light area as sensitizers may allow the reaction with onium salts [197,198,199].

### Two- or Three-Component Photoinitiating Systems Containing Iodonium Salt for Initiating Free Radical Photopolymerization Processes for an Obtained Dental Composites

The hydrophobicity of commonly used photoinitiating systems based on camphorquinone (CQ) and ethyl 4-(dimethylamino)benzoate (EDMAB) has limited their performance in the wet, oral environment. Therefore, to eliminate this limitation, a water-soluble iodonium salt is added mainly diphenyliodonium hexafluorophosphate (DPIHP) [129]. Iodonium salt as an accelerator in dental applications is usually found in a ternary initiating system containing CQ and a tertiary aromatic amine. However, it is also possible to use a two-component initiating system based on CQ and an iodonium salt (without a tertiary aromatic amine), except that, compared to the three-component system, slightly lower conversion rates are usually obtained.

In a two-component initiating system based on CQ/onium salt, after being irradiated with blue light, the exciplex state is formed; next, the onium salt is reduced by electron transfer. The resulting diphenyliodine free radical is unstable and quickly degrades to phenyliodine and phenyl free radical, which causes the reaction to be irreversible. These reactive phenyl forms are useful in initiating the photopolymerization. Radicals generated during polymerization propagation effectively cleave the C–I bond, releasing another radical and allowing the photopolymerization [200].

The three-component initiating system is usually based on CQ/aromatic amine/iodonium salt, and this system is characterized that the additional amine radicals are produced. In addition, CQ is regenerated through substitution of inactive and also termination radicals to active radicals in the form of phenyl radicals and the generation of positive active phenyl radicals [193].

This makes the photopolymerization process initiated by the ternary initiation system extremely efficient and fast. A similar degree of conversion and rate of polymerization compared to acylphosphine oxide (MAPO) or bis-acylphosphine oxide (BAPO) photoinitiators is even obtained [201,202,203]. 

The photoinitiating system based on CQ/iodonium salt or CQ/iodonium salt/amine have mainly found application in initiating traditional methacrylate monomers used in the production of dental composites [201,202,204,205,206,207]. The addition of iodonium salt to the photoinitiating systems used to prepare dental composites brings many benefits. The most important are the

- Increase conversion in short photo-activation time;
- Reduced inhibitory polymerization effect from an organic solvent;
- Improved dentin bonding performance;
- Improved reactivity and mechanical properties;
- Decreased sorption and water solubility;
- Reduced initial color and improved color stability.

Many publications have been reported about the beneficial effect of onium salts on the properties of the final dental product obtained by free radical photopolymerization [79,130,138,147,184,205,206,208,209,210,211,212,213,214,215,216]. Researchers from Brazil in 2007 showed that the addition of onium salt improves the polymerization kinetics in dental adhesive resin. The three-component photoinitiating system based on camphorquinone (CQ), ethyl 4-dimethylaminobenzoate (EDAB) and diphenyliodonium hexafluorophosphate (DPIHFP) showed an improvement on the polymerization rate of standard methacrylate monomers (bisphenol A glycidyl dimethacrylate (Bis-GMA), triethylene glycol dimethacrylate (TEGDMA) and 2-hydroxyethyl methacrylate (HEMA)), leading to high conversion of monomers in short photo-activation time [130]. In another study, the same science team demonstrated that onium salt reduces the inhibitors polymerization effect from ethanol in a model dental adhesive resin [214]. Four years later (in 2012), they proved that the same three-component photoinitiating system using iodonium salt showed similar microtensile bond strength to dentin when compared to the commercial light-cured binding system—Clearfil SE Bond (CSEB). Moreover, after one year of storage, dentin’s bond strength was higher for three-component initiating systems [205]. That the addition of iodonium salt in a photoinitiating ternary system combined with witch CQ and EDB increases the conversion of standard methacrylate monomers used in the production of dental composites and do not affect the dentin bond strength has also been confirmed in the work of Borges et al. [215]. They proved that iodonium salt increased conversion for the CQ-based system but had no significant influence on 1-phenyl-1,2-propanedione (PPD) or phenylbis (2,4,6-trimethylbenzoyl)-phosphine oxide (BAPO) systems. The fact that the addition of iodonium salt significantly improves the conversion rates for the CQ /EDAB/iodonium salt system and does not significantly increase the conversion rate for the PPD/EDAB/iodonium salt or BAPO/EDAB/iodonium salt systems results directly from the molar extinction coefficient values of photoinitiators. High extinction coefficients indicate a high probability of light absorption at a specific wavelength, leading to high quantum yields and overall conversion improvement [217]. As CQ has the lowest ε_λmax_ (~28 dm^3^/mol cm) compared to PPD (~150 dm^3^/ mol cm) and BAPO (~300 dm^3^/mol cm) [119] additional improvement the degree of the conversion provided by iodonium salt is more effective for CQ-system. The ternary system (BAPO + EDAB + iodonium salt) showed a slight increase degree of conversion in short photo-activation time compared to the binary BAPO/EDAB system. Nevertheless, it is extremely interesting that this ternary system showed significantly higher conversion rates (~7%) than the ternary photoinitiating system based on camphorquinone [147].

Iodonium salt can also be used as a component of photoinitiating systems containing thioxanthone. For example, the initiating systems in the form of 2-hydroxy-3-(3,4 dimethyl-9-oxo-9H-thioxanthen-2-yloxy)-*N,N,N*-trimethyl-1-propanaminium chloride (QTX), ethyl 4-dimethylaminobenzoate (EDAB), diphenyliodonium hexafluorophosphate (DPIHFP) and 2-hydroxy-3-(3,4 dimethyl-9-oxo-9H-thioxanthen-2-yloxy)-*N,N,N*-trimethyl-1-propanaminium chloride (QTX), diphenyliodonium hexafluorophosphate (DPIHFP) and p-toluenesulfonic acid sodium salt hydrate (SULF) are effective in initiating the process of radical photopolymerization of acrylate monomers used in dentistry. The use of these initiation systems leads to similar conversion rates as in the case of the standard two-component system (CQ + EDAB). Nevertheless, these systems showed lower reactivity [184].

The addition of iodonium salt to conventional initiating systems also improves the physical and mechanical properties of dental composites. Gonçalves et al. have reported that, with diligent use, a ternary photoinitiator system including camphorquinone (CQ), 2-dimethylamino)ethyl methacrylate (DMAEMA) and diphenyliodonium hexafluorophosphate (DPI) in BisGMA/TEGDMA in 1:1 mass ratio may improve not only reactivity but also the mechanical properties of dental resin without significantly increasing the polymerization stress [216]. This work demonstrates that the use of 0.5 mol% DPI showed the best balance between increasing photopolymerization kinetics and producing a polymer with appropriate physical properties. Moreover, C.R. Augusto et al. demonstrated that the addition of 0.5 mol% iodonium salt for commercially available dual-polymerizing self-adhesive resin types of cement-based on RelyX U100 (3M ESPE) and BisCem improved the physical properties of these materials, increasing the degree of conversion, microhardness and push-out bond strength [206]. In turn, Dressano et al. proved that methacrylate resin containing PPD + CQ with iodonium salt improved not only the conversion of the materials but also influenced their physicochemical properties positively. These systems had higher flexural strength and modulus of elasticity, cohesive strength, and lower sorption and water solubility [138]. Sauro et al. noticed that the inclusion of the hydrophilic ionic salt such as DPIHP increased the affinity between amphiphilic monomers (ethoxylated-Bisphenol-A-dimethacrylates and 2-hydroxyethyl methacrylate) and two-component photoinitiating system, enhanced the degree of conversion, glass transition temperature (Tg) and also resin permeability (rP) [208]. The research confirms that the introduction of an iodonium salt to the three-component initiator system based on CQ and aromatic amine or the two-component initiator system containing only CQ improves the aesthetic properties of the dental composite. Shin et al. proved that the introduction of iodonium salt affects to reduce initial color and improve color stability [79].

In 2016, Bouzrati-Zerelli et al. developed the new ternary system based on camphorquinone/triphenylgermanium hydride/iodonium salt as a powerful system for initiating photopolymerization of methacrylate monomers used in the production of dental composites in thin films or thick composites upon exposure to a dental blue LED centered at 477 nm. Higher conversion rates were recorded for this system than for the standard CQ/amine system. Excellent bleaching properties were also observed under irradiation in the presence of these photoinitiating system [209].

In turn, Kirschner et al. proposed completely new iodonium salts, iodonium sulfonates, as an amine replacement. PISs based on CQ/iodonium sulfonate presents an excellent performance in methacrylate monomers upon blue light irradiation, similar to the CQ/amine system. Moreover, particularly useful bleaching properties were obtained [210]. The same team in 2019 proposed another compound, aryliodonium ylides (AY), as high-performance iodonium salts and efficient additives to CQ/amine-based systems methacrylate polymerization under blue light. Arylyodonium ylides (Table 4) present a broadband of absorption spectra in the 300–400 nm region. This work demonstrates that enhanced polymerization performances were achieved for the CQ/amine/AY system compared to the reference CQ/amine system. Besides, these PISs showed good bleaching properties after polymerization, and interestingly, excellent initiating ability in strongly oxygen-inhibited conditions [211].

PISs based on camphorquinone (CQ)/sulfinate and CQ/sulfonate, with iodonium salt, are also proposed by Kirchner et al. [212]. These photoinitiating systems were compared to the traditionally CQ/amine system. They proved that sulfinates and sulfonates combination with CQ is a perfect alternative to the replacement of standard amines used in methacrylate dental resin. With the participation of these initiating systems, dental composites with excellent bleaching properties, color stability, and excellent mechanical properties were obtained. An interesting derivative of iodonium salt developed is also diphenyliodonium p-toluenesulfonate (DPIpTS) (Table 4). It is used in combination with camphorquinone as an amine replacement [212]. In turn new iodonium salt based on phosphine derivative propsed by Kirchner et al. exhibits two essential functions: phosphine moiety to overcome oxygen inhibition and an iodonium salt moiety as counter cation to initiate the polymerization process [213].

The structures of the tested compounds, composition, and the influence of iodonium salt on the properties of dental composites are presented in Table 4.

## 5. Iodonium Salts as Photoinitiators for Cationic and IPN Photopolymerization to Obtain a Dental Composites

In recent years, researchers from around the world have designed new initiating systems based on onium salts for cationic and thiol-ene photopolymerization processes for obtained dental composites.

In 2014, it was first described using composites based on cationic systems for dental applications [106]. It has been shown that the two-component system of the CQ/[4(1-methylethyl)phenyl][4-methylphenyl] iodonium tetrakis (pentafluorophenyl)borate, Rhodorsil 2074 is useful for initiating the cationic photopolymerization process of bis[2-(3,4-epoxycyclohexyl)ethyl]tetramethyldisiloxane, UV30. CQ promotes the photopolymerization process even in the absence of amines as a hydrogen donor. It can separate labile hydrogen from an epoxy monomer; carbon-concentrated radicals are formed, which are oxidized by onium salt. The complete conversion of the epoxy group was achieved after 50 s with blue irradiation.

Fu et al. proposed the use a three-component photoinitiation system comprising 1 wt.% CQ (camphorquinone), 2 wt.% DMAEMA (2-(dimethylamino) ethyl meth acrylate) and 2 wt.% diphenyliodonium hexafluorophosphate to initiate the copolymerization of the matrix resins which combine bisphenol-S-bis(3-methacrylate-2-hydroxy propyl) ether (BisS-GMA) with the expanding monomer unsaturated spiro orthoesters 2-methylene-1,4,6-trispiro[4,4] nonane (MTOSN), for minimizing the volumetric shrinkage that generally occurs during polymerization. The results supported that the dental composites based on the expanding monomer and three-component photoinitiator system engendered a more significant decrease of volumetric shrinkage and better mechanical properties [68].

Danso, R. et al. proposed new resins (Oxirane-Acrylate IPN System—OASys) based on p-Cycloaliphatic diepoxide EPALLOY 5000^TM^ (EP5000) and dipenta erythritol hexaacrylate (DPHA). A three-component initiating system in the form of (4-octyloxyphenyl)phenyliodonium hexafluoroantimonate (OPPI), CQ, and a co-reactant oligomeric diol 250 Mn poly(tetrahydrofuran) was used. These results demonstrate that OASys resins cure well, are more hydrophobic, and have lower shrinkage stress than BisGMA-based resins. However, they are mechanically weaker [218].

This new class of photoinitiators based on silyl glyoxylates to initiate cationic polymerization combined with an iodonium salt was presented in an article by Kirschner [179]. This system can be used to initiate free radical/cationic hybrid polymerization and for the synthesis of interpenetrating polymer networks. The system silyl glyoxylate/iodonium exhibits excellent polymerization performances and exceptional bleaching properties compared to other well-established photoinitiators (e.g., camphorquinone) [179]. This system is also suitable for initiating a hybrid monomer (2-vinyloxyethoxyethyl methacrylate [VEEM]). This monomer leads to a considerable improvement of the mechanical properties of the final polymer through hybrid polymerization [179].

Zang et al. proposed the use 1,2-diketone/iodonium salt (and optional NVK) systems to initiate cationic photopolymerization of epoxides or free radical photopolymerization of methacrylates. Most of the photoinitiating systems have exhibited higher initiation ability than the well-known CQ-based systems. Nevertheless, the study of the biocompatibility indicates that these materials exhibit cytotoxicity [219].

Summary of the photoinitiating systems consisting of iodonium salt for cationic and IPN photopolymerization used in a dental application is presented in Table 5.

## 6. Challenges of Photoinitiator Systems for Dental Applications, Future Trends and Practical Aspects

In recent years, several new initiating systems for dental composites have been developed. However, these are mainly photoinitiating systems used to obtain dental composites by radical photopolymerization [220]. Most of them have several of the significant disadvantages mentioned earlier in this article. Despite such significant progress, new initiating systems with improved properties are still being sought, mainly to produce dental composites obtained by cationic photopolymerization, which

- Are entirely safe for humans, eliminating the cytotoxic amines and acrylate monomers that often cause severe allergies;
- Do not generate yellow color-eliminating camphorquinone, greater aesthetics, and quality of the final product;
- Have better and/or comparable mechanical properties and, due to the use of polymerizable monomers via to the cationic mechanism, have reduced polymerization shrinkage;
- Is possible to be used with dental lamps emitting radiation in the visible light range for the curing process, eliminating harmful UV radiation.

## 7. Conclusions

In conclusion, it can be stated that in the scope of initiating systems for photocuring dental composites according to the radical mechanism [152], a significant milestone towards solutions guaranteeing the active initiation of this type of process has now been realized. In addition, in recent years, new initiating systems containing iodonium salt to initiate cationic and/or IPN photopolymerization processes have been developed. In this way, dental composites with better mechanical properties and reduced polymerization shrinkage were obtained. Nevertheless, in most cases, these are camphorquinone-containing systems that generate yellow color or toxic co-initiators. In addition, the complete elimination of acrylate monomers that often cause severe allergies is still a significant challenge for researchers.

The literature review has presented previous achievements in the field of radical photoinitiators dedicated to the preparation of dental composites; their advantages and disadvantages are discussed. The advantages of iodonium salts and their potential to initiate cationic photopolymerization processes of silorane monomers to obtain new-generation dental composites were also indicated.

## Figures and Tables

**Table 1:** Properties of the popular free radical monomers to obtained dental composites [84].

Monomer	Molecular Weight [g/mol]	ρ_mon_^a^ [g/cm^3^]	ρ_pol_^b^ [g/cm^3^]	ΔV_p_ [%]	Viscosity [mPa·s]
TEGDMA	286	1.072	1.250	−14.3	100
UDMA	470	1.110	1.190	−6.7	5000–10,000
Bis-GMA	512	1.151	1.226	−6.1	500,000–800,000

ρ_mon_^a^—density of monomer, ρ_pol_^b^—density of polymer.

**Table 2:** Summary of the photoinitiators used in dental application, their basic properties, and photoinduced cleavage of photoinitiators.

Acronym of Photoinitiator	Structure, Together with a Scheme of Photoinduced Cleavage of Photoinitiator	Maximum Absorbance/Characteristic of Absorbance	Advantages	Disadvantages	Ref.
CQ	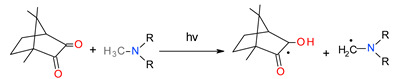	λ_max_ = 468nm	wide absorption range based on the visible range	molar extinction coefficient in the range of 400–500 nm is only 40 [dm^3^ · mol^–1^ · cm^–1^], strongly yellow color	[118,119]
PPD	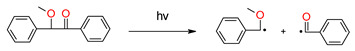	λ_max_ = 400nm	improve the color stability	necessary to use LED light sources with two violet emission bands (380–420 nm and blue 420–520 nm), dual peak LEDs	[136,138,139]
TPO	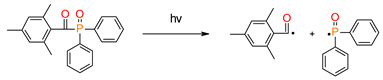	λ_max_ = 382nm	high efficiency of generating radicals, improve the color stability	low initiation efficiency, the need for UV light sources	[141,142,143]
IVO	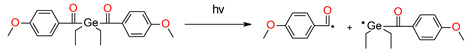	λ_max_ = 445nm	no cytotoxicity, high initiation rate and excellent bleaching	initiators only for free radical polymerization	[149,150,151]
HABI	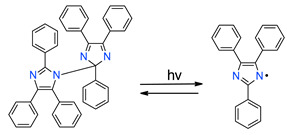	display extended absorption tails well into the visible spectral region	effective in initiating thiol-ene photopolymerization	poor absorption in the visible spectrum, sometimes requiring a photosensitizer, low solubility in standard resins used in a dental application and low solubility in organic solvents	[154,160,161]
Silane derivatives	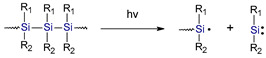	ultraviolent strong absorption in the 300–350 nm region	very effective in free radical photopolymerization, witch iodonium salts or pyridinium can be used for cationic photopolymerization	need for UV light sources	[162,163,164]
	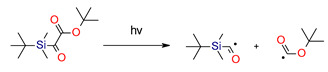	λ_max_ = 425 nm	excellent bleaching properties, in combination with an iodonium salt, can be useful for initiating cationic photopolymerization	low value of molar extinction coefficient ε: 120 dm^3^ · mol^–1^ · cm^–1^ in toluene and 100 dm^3^ · mol^–1^ · cm^–1^ in acetonitrile	[178,179]
	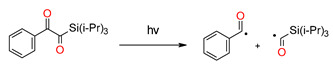	λ_max_ = 486nm for SED1 λ_max_ = 468 nm for SED2 in toluene	suitable for free radical photopolymerization exposure to blue (@ 455 nm) and even green (@ 520 nm) LED	not suitable for methacrylate photopolymerization	[180]
	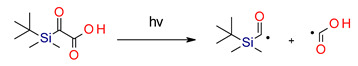	λ_max_ = 442 nm in acetonitirle	excellent bleaching properties, a high water solubility, and a very good stability in acidic	not suitable for methacrylate photopolymerization	[181]
TX	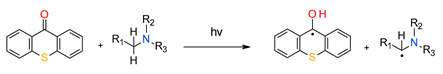	λ_max_ = ∼378 nm	water-soluble co-initiators, instant bond strength to dentin minimize the effects of concentration stress and phase separation in the aquatic environment	generally, less reactive than CQ/amine system	[151,182,183]

**Table 3:** Commercial iodonium photoinitiators used in industrial practice [196].

Photoinitiator	Structure	Wavelength of Maximum Absorbance (λ_max_) [nm]
Iodonium Photoinitiators Generating Benzene		
Hycure-810 (ChemFine)	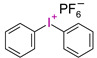	230–260 nm
Uvacure 1600 (Surface Specialties [UCB])	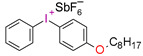	240 nm
Sarcat CD-1012 (Sartomer)	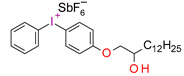	240 nm
Iodonium Photoinitiators not Generating Benzene		
OMNICAT 440 (IGM) or Hycure-820 (ChemFine)	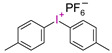	267 nm
Irgacure 250 (Ciba)	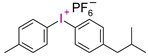	240–245 nm
UV 9310 (GE)	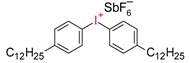	240 nm
Rhodorsil 2076 (Rhodia)	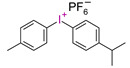	240 nm
Rhodorsil 2074 (Rhodia)	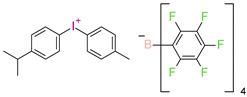	240–250 nm
Sylanto-7MP	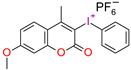	350 nm
Sylanto-7MS	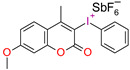	349 nm

**Table 4:**
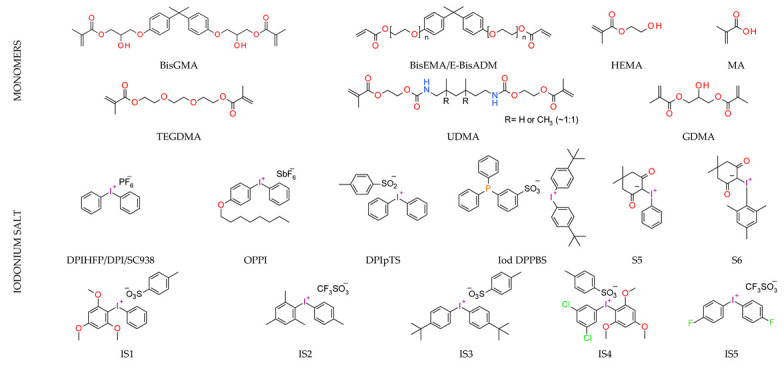

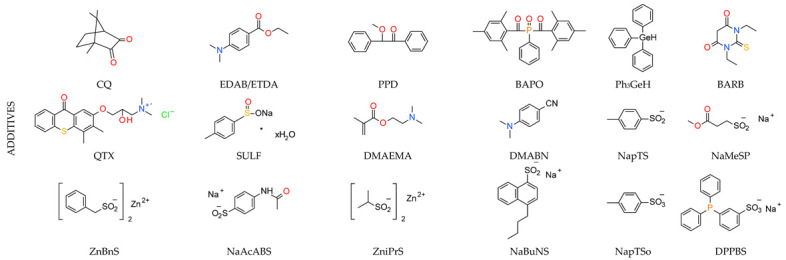
Summary of the photoinitating systems consisting iodonium salt for free radical photopolymerization used in dental application.

Photoinitiating Systems Based on Iodonium Salts	Reference of Photoinitiating System	Monomers/ Solution	Influence of Addition Iodonium Salt/Properties of the Dental Composition with Iodonium Salt	Ref.
1 mol% CQ + 0.25 mol% DPIHFP 1 mol% CQ + 0.5 mol% DPIHFP 1 mol% CQ + 1 mol% DPIHFP 1 mol% CQ + 2 mol% DPIHFP 1 mol% CQ + 4 mol% DPIHFP 1 mol% CQ + 2 mol% EDAB + 0.25 mol% DPIHFP 1 mol% CQ + 2 mol% EDAB + 0.5 mol% DPIHFP 1 mol% CQ + 2 mol% EDAB + 1 mol% DPIHFP 1 mol% CQ + 2 mol% EDAB + 2 mol% DPIHFP 1 mol% CQ + 2 mol% EDAB + 4 mol% DPIHFP	1 mol% CQ 1 mol% CQ + 0.25 mol% EDAB 1 mol% CQ + 0.5 mol% EDAB 1 mol% CQ + 1 mol% EDAB 1 mol% CQ + 2 mol% EDAB 1 mol% CQ + 4 mol% EDAB	50 wt.% Bis-GMA 25 wt.% TEGDMA 25 wt.% HEMA	increase conversion in short photo-activation time	[130]
1 mol% CQ + 1 mol% EDAB + 1 mol% DPIHFP	1 mol% CQ + 1 mol% EDAB	50 wt.% Bis-GMA 25 wt.% TEGDMA 25 wt.% HEMA (0, 10, 20, 30 and 40 wt.% ethanol)	reduce the inhibitory polymerization effect from an organic solvent	[214]
1 mol% CQ + 1 mol% DPIHFP 1 mol% CQ + 1 mol% EDAB + 1 mol% DPIHFP	1 mol% CQ 1 mol% CQ + 1 mol% EDAB	50 wt.% Bis-GMA 25 wt.% TEGDMA 25 wt.% HEMA	improve dentin bonding performance	[205]
1 mol% CQ + 1 mol% EDAB + 1 mol% DPIHFP 0.5 mol% CQ + 1 mol% EDAB + 0.5 mol% BAPO + 1 mol% DPIHFP 0.5 mol% CQ + 1 mol% EDAB + 0.5 mol% PPD + 1 mol% DPIHFP 1 mol% BAPO + 1 mol% EDAB + 1 mol% DPIHFP 1 mol% PPD + 1 mol% EDAB + 1 mol% DPIHFP	1 mol% CQ + 1 mol% EDAB 0.5 mol% CQ + 1 mol% EDAB + 0.5 mol% BAPO 0.5 mol% CQ + 1 mol% EDAB + 0.5 mol% PPD 1 mol% BAPO + 1 mol% EDAB 1 mol% PPD + 1 mol% EDAB	BisGMA:HEMA (60:40 wt.%) 20 wt.% ethanol	increase conversion, does not affect the dentin bond strength	[215]
1 mol% BAPO + 1 mol% DPIHFP 1 mol% BAPO + 2 mol% EDAB + 1 mol% DPIHFP 1 mol% CQ + 2 mol% EDAB + 1 mol% DPIHFP 1 mol% BAPO + 1 mol% CQ + 2 mol% EDAB + 1 mol% DPIHFP	1 mol% BAPO 1 mol% CQ 1 mol% BAPO + 2 mol% EDAB 1 mol% CQ + 2 mol% EDAB 1 mol% BAPO + 1 mol% CQ + 1 mol% EDAB	50 wt.% Bis-GMA 50 wt.% TEGDMA	highest polymerization and conversion rate for 1 mol% BAPO/1 mol% EDAB/1 mol% DPIHFP in short photo-activation time	[147]
QTX + DPIHFP QTX + EDAB + DPIHFP QTX + DPIHFP + BARB QTX + DPIHFP + SULF	QTX CQ + EDAB QTX + EDAB QTX + BARB QTX + SULF CQ + QTX + EDAB QTX + EDAB + BARB OTX + EDAB + SULF	50 wt.% Bis-GMA, 25 wt.% TEGDMA 25 wt.% HEMA	similar conversion rates as in the case of the standard two-component system (CQ + EDAB); lower reactivity	[184]
1 mol% CQ + 2 mol% DMAEMA/ 0.25, 0.5, 1, 2 or 4 mol% DPI	1 mol% CQ/2 mol% DMAEMA	20 wt.% Bis-GMA 20 wt.% TEGDMA 60 wt.% of silanated barium borosilicate glass fillers	improve the reactivity and mechanical properties	[216]
0.5, 1 or 2 mol% DPIHFP	commercially available dual-polymerizing self-adhesive resin cements: RelyX U100 (3M ESPE) and BisCem (Bisco Inc.)		increase the degree of conversion, microhardness and push-out bond strength	[206]
0.5 mol% CQ + 0.5 mol% DPI 1.0 mol% CQ + 0.5 mol% DPI 0.5 mol% PPD + 0.5 mol% DPI 1.0 mol% PPD + 0.5 mol% DPI 0.5 mol% CQ + 1 mol% DPI 1.0 mol% CQ + 1 mol% DPI 0.5 mol% PPD + 1 mol% DPI 1.0 mol% PPD + 1 mol% DPI 0.5 mol% CQ + 0.5 mol% PPD + 0.5 mol% DPI 0.5 mol% CQ + 1.0 mol% PPD + 0.5 mol% DPI 1.0 mol% CQ + 0.5 mol% PPD + 0.5 mol% DPI 1.0 mol% CQ + 1.0 mol% PPD + 0.5 mol% DPI 0.5 mol% CQ + 0.5 mol% PPD + 1 mol% DPI 0.5 mol% CQ + 1.0 mol% PPD + 1 mol% DPI 1.0 mol% CQ + 0.5 mol% PPD + 1 mol% DPI 1.0 mol% CQ + 1.0 mol% PPD + 1 mol% DPI	0.5 mol% CQ 1.0 mol% CQ 0.5 mol% PPD 1.0 mol% PPD 0.5 mol% CQ + 0.5 mol% PPD 0.5 mol% CQ + 1.0 mol% PPD 1.0 mol% CQ + 0.5 mol% PPD 1.0 mol% CQ + 1.0 mol% PPD	25 wt.% BisGMA 20 wt.% TEGDMA 10 wt.% GDMA 25 wt.% HEMA	improve flexural strength and modulus of elasticity, cohesive strength, as well as lower sorption and water solubility	[138]
0.25 wt.% CQ + 1 wt.% ETDA + 1 wt.% DPIHP	0.25 wt.% CQ + 1 wt.% ETDA	37 wt.% E-BisADM 25 wt.% TEGDMA 28% HEMA 10 wt.% ethanol 65 wt.% E-BisADM 25% TEGDMA 10 wt.% ethanol	enhance the degree of conversion, glass transition temperature (Tg) as well as resin permeability (rP).	[208]
1 part of CQ and 2 parts of OPPI Equally proportioned CQ, OPPI, and DMAEMA Total concentrations of 1 wt.% and 3 wt.%	CQ only 1 part of CQ and 2 parts of DMAEMA Total concentrations of 1 wt.% and 3wt.%	37.5 wt.% BisGMA 37.5 wt.% BisEMA 25 wt.% TEGDMA	reduce initial color and improve color stability	[79]
CQ + Ph_3_GeH + DPI in diffrent mass ratio	CQ + EDB + DPI CQ + EDB	70 wt.% Bis-GMA 30 wt.% TEGDMA 100 wt.% UDMA	excellent bleaching properties,	[209]
0.5 wt.% CQ + 1 wt.% IS1 0.5 wt.% CQ + 1 wt.% IS2 0.5 wt.% CQ + 1 wt.% IS3 0.5 wt.% CQ + 1 wt.% IS4 0.5 wt.% CQ + 1 wt.% IS5 0.2 wt.% CQ + 0.2 wt.% EDB +1 wt.% IS3	0.5 wt.% CQ + 1 wt.% EDB 0.2 wt.% CQ + 0.2 wt.% EDB 0.2 wt.% CQ + 0.2 wt.% EDB + 1 wt.% SC938	30 wt.% BisGMA 70 wt.% TEGDMA 10 wt.% MA 63 wt.% BisGMA 27 wt.% TEGDMA 10 wt.% HEMA 63 wt.% BisGMA 27 wt.% TEGDMA	excellent bleaching properties, very good performance,	[210]
0.2 wt.% CQ + 0.5 wt.% EDB + 1 wt.% S5 0.2 wt.% CQ + 0.5 wt.% EDB + 1 wt.% S6 1 wt.% CQ + 1 wt.% EDB + 1 wt.% S5 1.5 wt.% CQ + 0.6 wt.% DMABN + 0.75 wt.% S5 1.5 wt.% CQ + 0.6 wt.% DMABN + 0.75 wt.% S6	0.2 wt.% CQ + 0.5 wt.% EDB 0.2 wt.% CQ + 0.5 wt.% EDB + 1 wt.% SC938 1 wt.% CQ + 1 wt.% EDB 1.5 wt.% CQ + 0.6 wt.% DMABN	Spectrum^®^ TPH^®^3 resin received from Dentsply Sirona consisting of a mixture of modified BisGMA, TEGDMA and other methacrylate monomers 30 wt.% BisGMA 70 wt.% TEGDMA Prime&Bond Active^®^ resin	strongly oxygen-inhibited conditions, excellent bleaching properties	[211]
0.5 wt.% CQ + 1 wt.% NaMeSP + 1 wt.% SC938 0.5 wt.% CQ + 1 wt.% ZnBuS + 1 wt.% SC938 0.5 wt.% CQ + 1 wt.% NaAcABS + 1 wt.% SC938 0.5 wt.% CQ + 1 wt.% ZniPrS + 1 wt.% SC938 0.5 wt.% CQ + 1 wt.% NaBuNS + 1 wt.% SC938 0.5 wt.% CQ + 1 wt.% NapTSo + 1 wt.% SC938 0.5 wt.% CQ + 1 wt.% DPIpTS 0.2 wt.% CQ + 0.5 wt.% EDB + 1 wt.% NaMeSP+ 1 wt.% SC938 0.2 wt.% CQ + 0.5 wt.% EDB + 1 wt.% NapTSo+ 1 wt.% SC938 0.2 wt.% CQ + 0.5 wt.% EDB + 1 wt.% SC938	0.5 wt.% CQ + 1 wt.% NaMeSP 0.5 wt.% CQ + 1 wt.% ZnBuS 0.5 wt.% CQ + 1 wt.% NaAcABS 0.5 wt.% CQ + 1 wt.% ZniPrS 0.5 wt.% CQ + 1 wt.% NaBuNS 0.5 wt.% CQ + 1 wt.% NapTSo 0.5 wt.% CQ + 1 wt.% EDB 0.2 wt.% CQ + 0.5 wt.% EDB	30 wt.% BisGMA 70 wt.% TEGDMA 10 wt.% MA 63 wt.% BisGMA 27 wt.% TEGDMA 10 wt.% HEMA 63 wt.% BisGMA 27 wt.% TEGDMA	excellent bleaching properties, color stability, excellent mechanical properties	[212]
0.5 wt.% CQ + 1 wt.% DPPBS + 1 wt.% Iod 0.5 wt.% CQ + 1 wt.% Iod-DPPBS 0.4 wt.% CQ + 0.1 wt.% Iod-DPPBS 0.4 wt.% CQ + 0.4 wt.% Iod-DPPBS 0.4 wt.% CQ + 0.6 wt.% Iod-DPPBS 0.4 wt.% CQ + 1 wt.% Iod-DPPBS 0.5 wt.% CQ + 0.2 wt.% EDB + 1 wt.% DPPBS 0.5 wt.% CQ + 0.2 wt.% EDB + 1 wt.% DPPBS + 1 wt.% Iod 0.5 wt.% CQ + 0.1 wt.% EDB + 1 wt.% DPPBS + 1 wt.% Iod 0.5 wt.% CQ + 1 wt.% DPPBS + 1 wt.% Iod	0.5 wt.% CQ + 1 wt.% EDB 0.5 wt.% CQ + 0.2 wt.% EDB 0.4 wt.% CQ + 1 wt.% Iod 0.5 wt.% CQ + 0.2 wt.% EDB + 1 wt.% Iod	30 wt.% BisGMA 70 wt.% TEGDMA	oxygen inhibition	[213]

BisGMA—bisphenol glycidyl methacrylate; BisEMA/E-BisADM—ethoxylated Bisphenol A dimethacrylate; HEMA—2-hydroxyethyl methacrylate; TEGDMA—triethyleneglycol dimethacrylate; UDMA—urethane dimethacrylate; GDMA—1,3-glycerol dimethacrylate; DPIHFP/DPI/SC938—diphenyliodonium hexafluorophosphate; OPPI—p-octyloxy-phenyl-phenyl iodonium hexafluoroantimonate; DPIpTS—diphenyliodonium p-toluenesulfinate; S5—aryliodonium ylides; S6—aryliodonium ylides; IS1—phenyl(2,4,6-trimethoxyphenyl)iodonium p-toluenesulfonate; IS2—(4-methylphenyl)(2,4,6-trimethylphenyl)iodonium trifluoromethanesulfonate; IS3—bis(4-tert-butylphenyl) iodonium p-toluenesulfonate; IS4—3,5-dichlorophenyl)(2,4,6-trimethoxyphenyl)iodonium p-toluenesulfonate; IS5- bis(4-fluorophenyl)iodonium trifluoromethanesulfonate (IS5); CQ—camphorochinone; EDAB/ETDA—dimethylaminoethyl amine benzoate; PPD—1-phenyl-1,2-propanedione; BAPO—phenylbis (2,4,6-trimethylbenzoyl)-phosphine oxide; Ph_3_GeH—triphenylgermanium hydride; BARB—1,3-diethyl-2-thio-barbituric acid; QTX—2-hydroxy-3-(3,4dimethyl-9-oxo-9H-thioxanthen-2-yloxy)-N,N,N-trimethyl-1-propanaminium chloride; SULF—p-toluenesulfinic acid sodium salt hydrate; DMAEMA—2-dimethylaminoethyl methacrylate; NapTS—sodium p-toluenesulfinate; NaMeSP—sodium 1-methyl 3- sulfinopropanoate; ZnBnS zinc benzylsulfinate; NaAcABS—sodium 4-(acetylamino)benzenesulfinat; ZniPrS—zinc isopropylsulfinate; NaBuNS—sodium butylnaphtalenesulfinate; NapTSo—sodium p-toluenesulfonate; DPPBS—sodium 3-(diphenylphophino)benzenesulfonate.

**Table 5:** Summary of the photoinitiating systems consisting of iodonium salt for cationic and IPN photopolymerization used in a dental application.

Iodonium Salt	Other Co-initiators		Monomers		Properties	Ref.
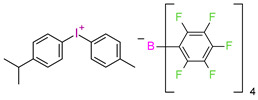	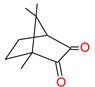	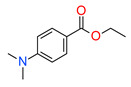	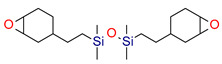		lowering of shrinkage	[106]
Rhodosil 2074	CQ	EMBO	UV 30			
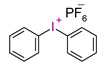 DPIHFP	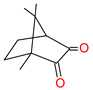		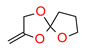		greater decrease of volumetric shrinkage and better mechanical properties	[68]
	CQ		MTOSN			
	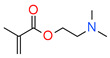		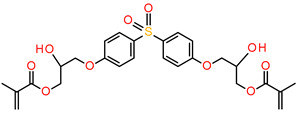			
	DMAEMA		BisS-GMA			
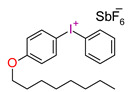 OPPI	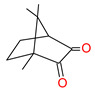		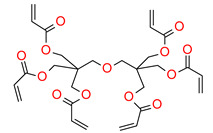		lower shrinkage stress more hydrophobic mechanically weaker	[218]
	CQ		DPHA			
	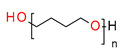		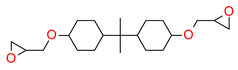			
	Diol		EPS5000			
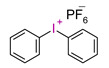	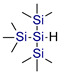		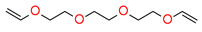	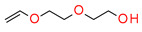	exceptional bleaching properties better mechanical properties	[179]
DPI	(TMS)_3_SiH		DVE-3	DEGVE		
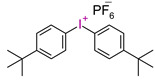	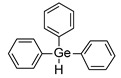		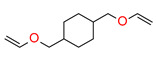	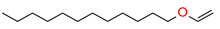		
SC938	Ph_3_GeH		CHDVE	DODECYL VINYL ETHER		
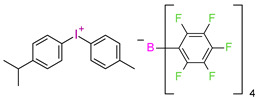	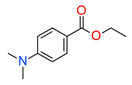		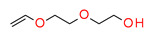	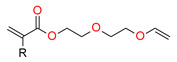		
PI 2074	EDB		DEGDVE	VEEM		
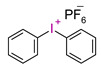 IOD	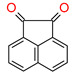		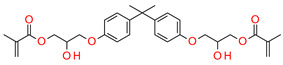		higher initiation ability than the well-known CQ-based systems, but cytotoxicity	[219]
	ANPQ		BisGMA			
	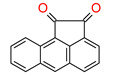		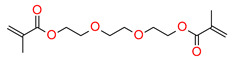			
	AATQ		TEGDMA			
	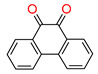		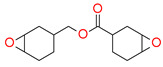			
	PANQ		EPOX			
